# Hierarchical Clustering and Target-Independent QSAR for Antileishmanial Oxazole and Oxadiazole Derivatives

**DOI:** 10.3390/ijms23168898

**Published:** 2022-08-10

**Authors:** Henrique R. Teles, Leonardo L. G. Ferreira, Marilia Valli, Fernando Coelho, Adriano D. Andricopulo

**Affiliations:** 1 Laboratory of Medicinal and Computational Chemistry (LQMC), Center for Research and Innovation in Biodiversity and Drug Discovery (CIBFar), Institute of Physics of São Carlos, University of São Paulo (USP), Av. João Dagnone, n° 1100, São Carlos 13563-120, SP, Brazil; 2 Laboratory of Synthesis of Natural Products and Drugs, Institute of Chemistry, University of Campinas, Campinas 13083-970, SP, Brazil

**Keywords:** *Leishmania infantum*, visceral leishmaniasis, QSAR, medicinal chemistry

## Abstract

Leishmaniasis is a neglected tropical disease that kills more than 20,000 people each year. The chemotherapy available for the treatment of the disease is limited, and novel approaches to discover novel drugs are urgently needed. Herein, 2D- and 4D-quantitative structure–activity relationship (QSAR) models were developed for a series of oxazole and oxadiazole derivatives that are active against *Leishmania infantum*, the causative agent of visceral leishmaniasis. A clustering strategy based on structural similarity was applied with molecular fingerprints to divide the complete set of compounds into two groups. Hierarchical clustering was followed by the development of 2D- (*R*^2^ = 0.90, *R*^2^pred = 0.82) and 4D-QSAR models (*R*^2^ = 0.80, *R*^2^pred = 0.64), which showed improved statistical robustness and predictive ability.

## 1. Introduction

Visceral leishmaniasis (VL) is a neglected tropical disease (NTD) caused by the protozoan parasites *Leishmania infantum* and *L. donovani* and is the most severe form of leishmaniasis. Transmission occurs through the bite of infected female Phlebotominae sandflies. VL is a fatal disease if not treated and is the second leading cause of death among parasitic conditions after malaria. The disease has become a severe global health problem, with more than 200 million people currently at risk of infection worldwide [1]. The current treatments include drugs such as amphotericin B, sodium stibogluconate, miltefosine, and paromomycin, which present several drawbacks, including distribution and availability issues, long-term and complex treatment regimens, teratogenicity, toxicity, and drug resistance [2,3]. These shortcomings, combined with the high burden caused by the disease, highlight an urgent need for new treatment options for VL.

Despite the widespread use of the available drugs, little is known about their mechanisms of action, which reflects the main strategy that has been used for NTD drug discovery: phenotypic screening [4]. Despite the lack of information on the molecular targets, the phenotypic strategy is useful to account for activity against whole cells along with aspects of cell uptake, cytocidal or cytostatic mechanisms, and time-to-kill, among other relevant issues.

Therefore, the phenotypic approach has been widely used in drug discovery for NTDs given the very few validated molecular targets explored in the field [5,6].

Aligned with the strategy of phenotypic screening, the combination of different bioactive chemical scaffolds can be used to enrich the chemical diversity explored in NTD drug discovery. Recently, our group reported a hybridization approach combining two heterocyclic cores presenting antiparasitic activity, which guided the design of novel hybrid compounds with promising antileishmanial and anti-Trypanosoma cruzi activities [4]. One core is represented by oxadiazoles, and the other is 3-substituted 2-oxindoles, both of which are useful scaffolds, especially as antileishmanial agents [7,8,9,10,11].

The main goal of this work was the development of QSAR models using our in-house set of oxazoles and oxadiazoles that displayed in vitro antileishmanial activity against intracellular amastigotes. To this end, a computational approach using 2D- and 4D-QSAR strategies was conducted. Hierarchical clustering based on ligand structure was applied to split the dataset into two structurally similar groups. The integration of the experimental results with the applied ligand-based drug design studies (LBDD) yielded statistically significant QSAR models with the ability to predict the activity of new antileishmanial agents within a defined applicability domain.

## 2. Results and Discussion 

### 2.1. AutoQSAR

The initial model was obtained considering the complete set of molecules using the AutoQSAR method. A set of seven binary fingerprints (dendritic, linear, atom pair, atom triplet, topological, MOLPRINT 2D, and radial) was generated to characterize the structures and create the 2D molecular descriptors. Different regression techniques, such as multiple linear regression (MLR), partial least squares regression (PLS), principal components regression (PCR), and kernel-based PLS (KPLS), were adopted to build the set of models. Random selection of the molecules for the test and training sets was applied using the AutoQSAR machine learning routine specifically designed for this end. All seven binary fingerprints available were used as molecular descriptors to build the 2D-QSAR models. The fingerprints and regression approaches were systematically combined to generate the best models, which are described in Table 1.

For the complete dataset, the model that produced the best statistical parameters and score was obtained by the MOLPRINT2D binary fingerprint. The best regression method selected by the AutoQSAR routine was the KPLS technique with an 80:20 training/test set ratio, that is, 52 molecules in the training set and 13 compounds in the test set. This model yielded an *R*^2^ value of 0.6304 and a *Q*^2^ value of 0.6107.

Aiming to improve the predictive ability of the model, a structural analysis of the molecules in the dataset suggested that they have a diverse scaffold pattern. Therefore, to improve the QSAR results, hierarchical clustering was applied to the entire dataset.

**Figure 1:**
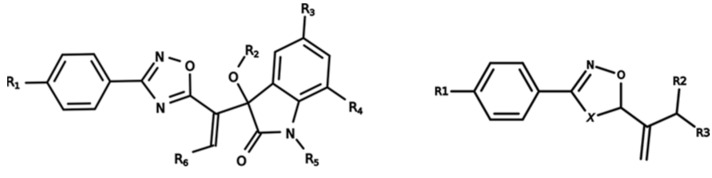
General scaffold of group G_1_ (**left**) and group G_2_ (**right**).

**Figure 2:**
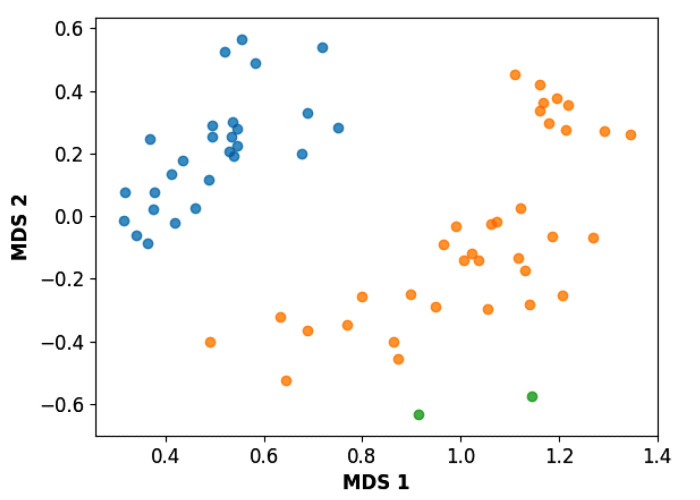
Scatter plot obtained by multidimensional scaling (MDS). The molecules of the G_1_ group are shown in blue, the molecules of the G_2_ group are shown in orange, and the structural outliers are shown in green.

To start the hierarchical clustering analysis, the binary fingerprints (dendritic, linear, atom pair, atom triplet, topological, MOLPRINT 2D, and radial) were calculated for the entire set of molecules and used as molecular descriptors for this analysis. First, the Kelley level [12] was used to select the optimal number of clusters. In this step, a considerable number of singletons (clusters with one molecule) were obtained, which indicates the structural diversity in the dataset. The next step was the separation of the entire dataset into two groups. To separate these two groups according to the similarity between the molecules, the total number of clusters was divided to generate only two clusters which were as populous as possible; i.e., starting with the Kelley level, the number of clusters was reduced until the formation of two groups. These two groups of compounds were defined so that both groups included a considerable number of molecules to build the two QSAR models. This strategy resulted in the exclusion of two molecules that were identified as structural outliers [13,14]. As a result of this cluster analysis, the initial dataset originated two groups of compounds: the G_1_ group with 27 compounds and the G_2_ group with 35 compounds. The scaffolds of each group are presented in Figure 1. The structural diversity present in the dataset can be observed through the scatter plot obtained with the multidimensional scaling (MDS) plot illustrated in Figure 2. From each of these groups, two new and independent QSAR models were built.

It is worth noting that group G_1_ in Figure 1 and Figure 2 is less structurally diverse than group G_2_. In Figure 2, the molecules of the G_1_ group are more concentrated, while in the G_2_ group, the molecules are more dispersed over the MDS plot. This structural diversity may have influenced the QSAR statistical parameters for each group, as the less diverse group (G_1_) resulted in better statistical indicators.

For the G_1_ group, the model that produced the best statistical parameters and score was represented by radial fingerprints and included 22 molecules in the training set and 5 in the test set (proportion 80:20). This is indicated by *R*^2^ = 0.9069, SD = 0.1039, *Q*^2^ = 0.8201, RMSE = 0.0945 and KPLS factor = 2. The best models for each training/test set split are shown in Table 2.

For the G_2_ group, the model that produced the best statistical parameters was represented by dendritic fingerprints and included 28 molecules in the training set and 7 in the test set (proportion 80:20). This finding is indicated by *R*^2^ = 0.8206, SD = 0.1377, *Q*^2^ = 0.8001, RMSE = 0.1081 and KPLS factor = 3. The best models for each split are shown in Table 3.

**Figure 3:**
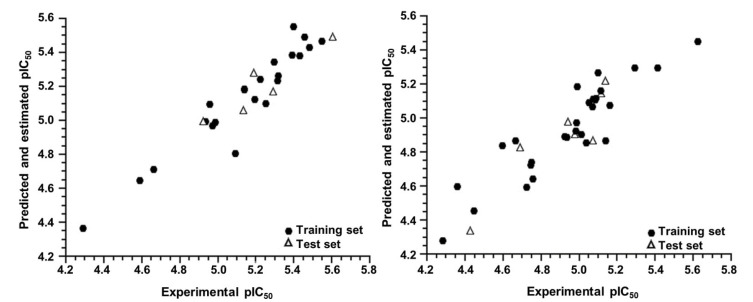
Experimental versus predicted and estimated pIC_50_ values for the training and test sets of the G_1_ (**left**) and G_2_ (**right**) groups.

The predicted pIC_50_ values obtained for both groups, G_1_ and G_2_, are represented graphically in Figure 3 along with the experimental pIC_50_ values. Both plots show good agreement between the experimental and predicted activity for the AutoQSAR models. In addition, Table 4 shows the predicted and experimental pIC_50_ values for the entire dataset (complete set model) and for the two groups of molecules obtained after the hierarchical cluster analysis (cluster model).

By examining the scaffolds of the two groups, it is noticeable that although the structures of the molecules of both clusters have similarities, the merging of the two groups, which would represent greater structural diversity, does not result in improved models. In line with this finding, the G_2_ group, which has a greater structural diversity, represented by a smaller scaffold and larger R substituents, demonstrated a slightly smaller improvement than G_1_ in the statistical parameters (a difference of 0.0863 in *R*^2^ and 0.02 in *Q*^2^).

**Figure 4:**
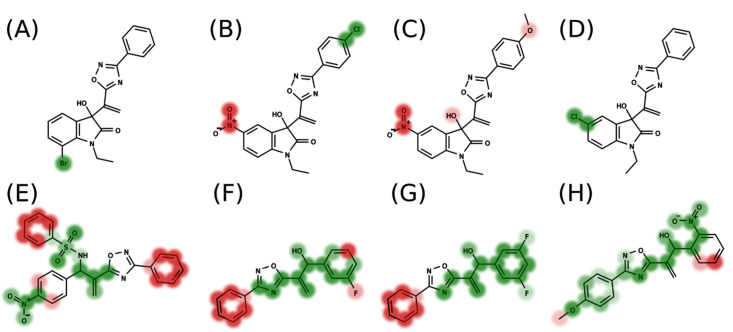
Contribution map generated by KPLS of groups G_1_ (**A**–**D**) and G_2_ (**E**–**H**). Green colors represent positive contributions, and red colors indicate negative contributions to biological activity.

In addition to the predictive capacity, KPLS 2D-QSAR allows the visualization of regions in the molecules responsible for increasing or decreasing the biological response through the generation of contribution maps, as depicted in Figure 4. Green and red colors represent positive and negative contributions to response, respectively. For the G_1_ group, halogen substituents showed positive contributions to substituents R_1_, R_3_, R_4_ and R_5_. The only difference among molecules **7**, **22**, and **23** is the substitution of bromine, fluorine, and chlorine in substituent R_3_, and all substitutions in this position showed positive contributions. However, the chlorine in molecule **23** contributed to a greater increase in activity, which can be validated directly by the pIC_50_ in Table 4. In general, the nitro group substitution was unfavorable for molecules in G_1_. For the G_2_ group, the hydrogen atom in position R_1_ showed an unfavorable contribution. In this case, the phenyl group, and especially the methoxy group substitution, showed a favorable contribution in R_1_. An exception for the positive contribution of the nitro group was observed with the oxazole core. In substituent R_3_, halogen (except bromide) and nitro substituents showed a positive contribution, but asymmetrical electron density was slightly unfavorable, which favors double halogen substitutions in both meta positions or one halogen in the para position. For substituent R_2_, the hydroxyl group showed a positive contribution, while the benzene sulfonamide showed a negative contribution.

**Figure 5:**
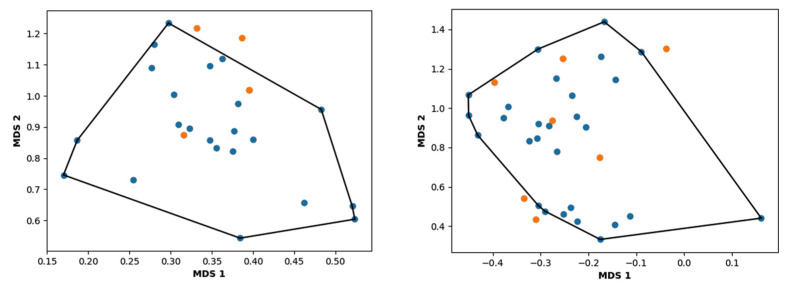
Applicability domain for group G_1_ (**left**) and group G_2_ (**right**). The molecules of the training set are illustrated in blue, and the compounds of the test set are depicted in orange.

The MDS scatter plot of the dataset, obtained by the geometric convex-hull method, allows the definition of the chemical space over which the model, represented by the training set, is applicable. The applicability domain for the 2D-QSAR models for the G_1_ and G_2_ groups is shown in Figure 5.

### 2.2. 4D-QSAR

Three-dimensional quantitative structure–activity relationship (3D-QSAR) modeling is a broadly used method in computer-assisted molecular design. The method assumes that changes in the binding affinities of ligands are related to changes in molecular properties represented by molecular fields. A common and popular method is comparative molecular field analysis (CoMFA). Some issues are inherent in 3D-QSAR [15,16], mainly in receptor-independent 3D-QSAR (RI-3D-QSAR). For example, the QSAR model in the CoMFA method is strongly dependent and sensitive to conformations and alignments of the molecules. Another limitation is that the bioactive conformation of a molecule should be used, which may not coincide with the lowest energy conformations, which are commonly used whenever the molecular target is unknown. In this work, several structural alignment methods were used as attempts to achieve a suitable alignment of the compounds to be used in subsequent CoMFA analyses. However, due to the limitations described above, no suitable CoMFA models were obtained (see Supplementary File for CoMFA models). Following these concepts, a 4D-QSAR approach was used in this work to address the limitations associated with 3D-QSAR models. The LQTA-QSAR approach explores the main advantages of both CoMFA and 4D-QSAR modeling [16,17]. This method is based on the generation of a CEP for each compound instead of only one conformation, which is followed by the calculation of 3D descriptors using the Coulomb and Lennard–Jones potentials. To generate the 4D-QSAR models, the strategy used in the 2D-QSAR analyses was repeated, i.e., we built a model with the entire dataset, which was followed by the generation of groups by using hierarchical clustering. Two-hundred training and test sets were randomly divided and subjected to QSAR model construction. For the 2D-QSAR studies, the best results were obtained by using an 80:20 ratio between the training and test sets; thus, the same ratio was applied to the 4D-QSAR analyses.

The model generated using the complete dataset was used for comparison purposes. LQTAgridPy software was used to generate a matrix with 21,252 descriptors. After applying a variance cutoff and the Pearson cutoff, 554 descriptors were subjected to PyQSAR. This software uses a clustering method to reduce the search space. In this step, PyQSAR also eliminates descriptors with low variance. A selection based on a genetic algorithm (GA) was used to maintain the best descriptors from the different clusters. The GA-based selections were repeated until the optimal variable selection was achieved. PyQSAR selected a set of descriptors that resulted in the following parameters: *R*^2^ = 0.4599, *R*^2^_pred =_ 0.4353. The set of selected descriptors included [15_19_20_NH3+_C], [15_20_15_NH3+_LJ], [16_21_20_NH3+_C], [16_23_10_NH3+_LJ], and [18_19_11_NH3+_C].

*Group* G_1_: The dataset used in the 4D-QSAR for G_1_ was the same as that previously used in the 2D-QSAR, with 27 compounds divided into 22 molecules for the training set and 5 compounds for the test set. The LQTAgridPy software resulted in a matrix with 19,404 descriptors. After truncation of the Lennard–Jones potential, the variance cutoff, and the Pearson cutoff, the filters led to a significant variable reduction to 903 descriptors. Each of these descriptors represents a grid point with the fields acting upon it. This reduced matrix was used as the input for the selection of variables and generation of the model by PyQSAR. The model chosen is represented by 5 descriptors (Equation (1)) and generated the following results: *R*^2^ = 0.8033, RMSE = 0.1313, *Q*^2^_5-fold =_ 0.6600, RMSE_cv =_ 0.1716, *R*^2^_pred =_ 0.6480.

pIC_50_ = 5.1535 + 0.8409[15_13_6_NH3+_LJ] −0.7075[16_12_5_NH3+_LJ] +0.1484[16_20_10_NH3+_LJ] −0.1210[21_17_13_NH3+_LJ] +0.1913[22_12_12_NH3+_LJ](1)

*Group* G_2_: For G_2_, the same 35 compounds used in 2D-QSAR, divided into 28 compounds in the training set and 7 in the test set, were employed in the 4D-QSAR. The LQTAgridPy software resulted in a matrix with 21,252 descriptors. After truncation of the Lennard–Jones potential, the variance cutoff, and the Pearson cutoff, the filters led to a significant variable reduction to 3353 descriptors. Each of these descriptors represents a grid point with the fields acting on it. This reduced matrix was used as the input for the selection of variables and generation of the model by PyQSAR. The selected model is represented by five descriptors (Equation (2)) and generated the following results: *R*^2^ = 0.7005, RMSE = 0.156, *Q*^2^_5-fold =_ 0.6095, RMSE_cv =_ 0.1701, *R*^2^_pred =_ 0.6581.

pIC_50_ = 4.9338 + 0.2170[16_20_11_NH3+_LJ] +0.1303[17_19_15_NH3+_LJ] −0.7328[17_26_15_NH3+_C] +0.2770[18_23_14_NH3+_LJ] +0.7227[19_26_20_NH3+_C](2)

The 4D-QSAR statistical parameters are summarized in Table 5.

**Figure 6:**
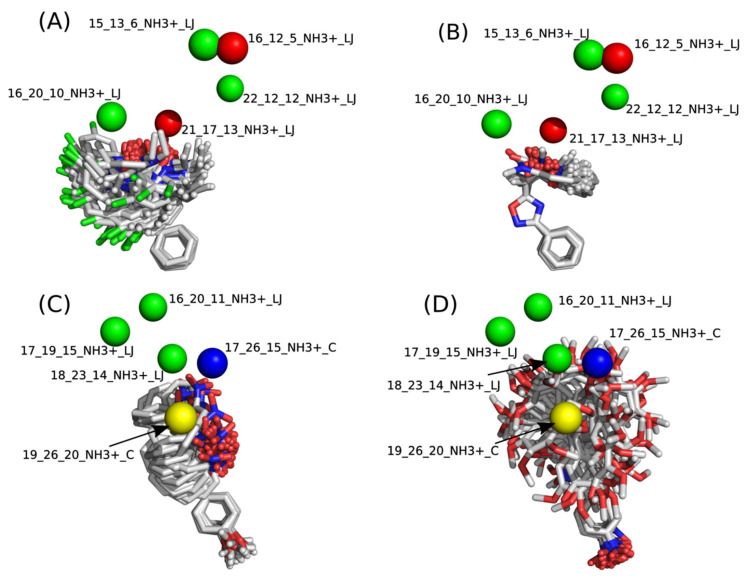
Contribution maps of the most and least potent compounds of group G_1_ (**A**,**B**) and group G_2_ (**C**,**D**).

The contribution maps were generated to allow the visualization of the positive and negative contributions of groups in the 4D-QSAR model (Figure 6). Green spheres represent steric interactions with positive regression coefficients, and red represents steric interactions with negative regression coefficients. Similarly, blue spheres indicate electrostatic descriptors with negative regression coefficients, and yellow represents positive regression coefficients. Positive coefficients contribute positively to the pIC_50_ values, while negative coefficients contribute negatively. The analysis for both groups G_1_ and G_2_ indicates that the major correlation between structure and activity is not related to the oxadiazole or oxazole core, but primarily to the substituents attached to these rings.

*Group* G_1_: For G_1_, the positive steric contributions [16_20_10_NH3+_LJ] and [22_12_12_NH3+_LJ] are mainly related to the halogen substituents at R_4_ and R_5_, which, due to energy minimization, are facing towards [16_20_10_NH3+_LJ] or [22_12_12_NH3+_LJ] in some of these molecules. The negative steric contribution [21_17_13_NH3+_LJ] is related to the hydroxyl group, and mainly to bulky substituents at R_2_. The results of the 4D-QSAR models for G_1_ are related to the degree of flexibility and pIC_50_. Molecules with a higher degree of freedom showed poor biological activity, which may pose an obstacle to the formation of stable intermolecular interactions with the molecular target [18].

*Group* G_2_: For G_1_, a higher conformational degree of freedom of the molecules is also associated with low pIC_50_ values, which can be noticed in Figure 6 when comparing the least and most active compounds. The descriptor [18_23_14_NH3+_LJ] indicates that bulky substituents at group R_3_, mainly represented by compound **31** with an ethenylbenzene (styrene) substituent, show a positive steric contribution. The positive contribution of [19_26_20_NH3+_C] is associated with halogen-substituted compounds in the para position of the phenyl in group R_4_, whereas the [17_26_15_NH3+_C] contribution indicates that these same atoms can decrease the biological response because of the assumed conformations.

## 3. Materials and Methods

### 3.1. Dataset Characterization

**Figure 7:**
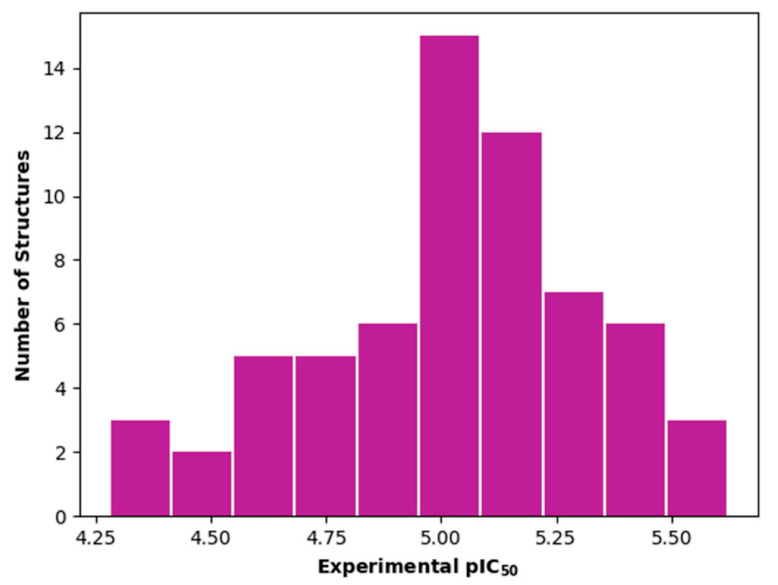
Histogram for the distribution of the experimental pIC_50_ values over the entire compound set used in the QSAR studies.

The dataset used for both QSAR modeling methods includes 64 molecules, 62 having an oxadiazole ring and 2 having an oxazole core, as shown in Table 6. The in vitro assays against *L. infantum* were performed in our research group using the same experimental conditions, as previously reported [4]. The potency of the compounds was expressed as the concentration required to kill 50% of parasites in vitro (IC_50_). The antileishmanial activity was determined as the number of intracellular amastigotes in THP-1 macrophages, which is the relevant form of the parasite for drug discovery purposes. The IC_50_ values (ranging from 2.38 to 52.59 µM) were converted into pIC_50_ values for appropriately scaling the data, which ranged from 4.28 to 5.62. The distribution of pIC_50_ values over the dataset compounds is illustrated in the histogram in Figure 7.

In addition to the characterization of the activity profile of the dataset, a scaffold analysis was performed for the R-groups using Canvas (Maestro, Schrödinger) [19]. The general scaffolds for the series were generated through an automated search for the maximum common substructure (MCS).

### 3.2. 2D-QSAR

The 2D-QSAR was performed with the machine learning tools of AutoQSAR [19] embedded in Maestro [20] (release 2016-3, Schrödinger LLC, New York, NY, USA) as previously reported [21,22]. In all AutoQSAR calculations, the proportion between the test and training sets was defined as follows: 70:30 (70% of the compounds for training the models and 30% for the test set), 75:25, and 80:20. The best model was selected based on internal validation parameters, such as the regression coefficient (*R*^2^) and the 8 standard deviation (SD) for the training set, and external validation parameters, i.e., the predicted regression coefficient (*Q*^2^) and the root-mean-square error (RMSE) for the test set compounds. The best models were recreated within Canvas using the same test set, training set, and binary fingerprint generated in the AutoQSAR modeling.

### 3.3. Hierarchical Clustering

Hierarchical clustering based on 2D similarity analyses of the dataset was performed using Canvas 1.1 software. Linear fingerprints were calculated, and the similarity matrix was evaluated using the Tanimoto coefficient as the similarity metric [23]. This is a popular similarity metric for comparing chemical structures represented by means of fingerprints, and structures are usually considered similar if the index is higher than 0.85. A higher number of shared features results in an index closer to 1. Conversely, a higher number of unique features results in an index closer to zero. The agglomerative method chosen was the average linkage. Based on the similarity results, the dataset compounds were split into two groups, and the Kelley index was used to select the optimal number of clusters [12]. Following this procedure, the number of clusters was decreased to generate two different groups so that if the number of clusters was reduced again, it would result in the merging of groups G_1_ and G_2_. To visualize the diversity of the identified groups in a plane, a multidimensional scaling (MDS) approach was employed with the similarity matrix as input in a Knime node [24].

The applicability domain (AD) defines a region or limits where the model is able to reliably perform according to predictions [25]. The AD generated in this work was built using the geometric convex-hull method [26]. After the MDS, the coordinates of the training set were submitted to SciPy to generate the convex-hull output.

### 3.4. 4D-QSAR

The 4D-QSAR was performed with the LQTA-QSAR method [27]. The molecular dynamics simulation was performed using GROMACS version 4.6.5 [28,29]. A dodecahedron box was filled with explicit transferable intermolecular potential 3-point (TIP3P) water molecules, and the ffG43a1 [30,31] force field was used for the all-atom molecular simulations. The minimum distance between the molecule and walls was set to 10 Å. The energy minimization step was performed using the steepest descent gradient and conjugate gradient methods for a maximum of 4000 calculation steps. The pressure of the system was controlled by Parrinello–Rahman [32] coupling, and the temperature was kept constant by the Berendsen thermostat [33]. The volume of the system was balanced by heating in steps of 50 K, 100 K, 200 K, and 350 K for 10 ps each, and the system was ultimately cooled to 300 K for a 500 ps simulation. All the conformations for each ligand obtained through molecular dynamics simulations were placed in a “.gro” file extension. The conformational ensemble profile (CEP) to be used for the 4D-QSAR models was assembled considering the ligand conformations obtained from 50 to 500 ps. The alignment was generated considering the matching of the atom positions of the oxazole and oxadiazole rings. The alignment was submitted to LQTAgridPy, a Python version of LQTAgrid. The probe NH^+3^ was selected and used to represent the N-terminal unit. The probe swept all grid points from the box to compute all Coulomb and Lennard–Jones descriptors. The data were preprocessed with the energy cutoff of the Lennard–Jones descriptors from the CoMFA method. If the descriptor computed at an x, y, z position had a value of Lennard–Jones energy equal to or lower than 30 kcal/mol, no cutoff was applied. Otherwise, if the energy value exceeded 30 kcal/mol, then the logarithmic value of the residual was added to 30 kcal/mol, according to the following:

LJ_x,y,z_ < 30 kcal/mol → LJ_x,y,z_ = LJ_x,y,z_ LJ_x,y,z_ ≥ 30 kcal/mol → L J_x,y,z_ = 30 + logLJ_x,y,z_ − 30

The filtering method for the descriptor selection excluded those variables with absolute values of the Pearson correlation coefficient (|r|) of less than 0.2 with respect to the pIC_50_ [18,27] and the low-variance descriptors that only slightly changed between compounds (those with variance below the cutoff value of 0.01). The remaining descriptors were selected by PyQSAR [34], an open-source QSAR model generator. The variable selection used in PyQSAR uses the strategies of hierarchical clustering and a genetic algorithm (GA). Finally, multiple linear regression (MLR) was performed with the generated descriptors, and the pIC_50_ values were used as the independent variables to build the model. The process of internal validation was carried out through conventional noncross-validated correlation (*R*^2^). The robustness was examined by 5-fold cross-validated correlation (*Q*^2^_5-fold_) coefficients. For external validation, the test set was evaluated according to the coefficient of determination of external validation (*R*^2^_pred_). The images of the contribution maps were created by using PyMOL version 1.8.4.0 [35].

## 4. Conclusions

Receptor-independent QSAR methods were employed in the development of 2D- and 4D-QSAR models for a series of oxadiazole and oxazole antileishmanial derivatives. The clustering of the dataset proved to be advantageous for optimizing the statistical parameters in both the 2D-QSAR and 4D-QSAR models presented in this work. The final models exhibited good internal consistency and external predictive power and were able to accurately predict the pIC_50_ values when compared to the experimental values for both 2D and 4D models within the applicability domain. Once new compounds are designed, the hierarchical clustering, MDS plot, and applicability domain are useful tools to evaluate which group they belong to, and then the corresponding model can be applied. The results for the 2D-QSAR models compared to that of the 4D models suggest that for this dataset, 2D descriptors correlate better to the variation in the biological activity. The reasons for poorer results in QSAR methods that require 3D conformations are unknown; however, they may be linked to the mode of action of this series, which is yet to be discovered. Although the molecular target of these compounds is so far unknown, we can speculate from the structure of the compounds that the several functionalities that are able to form hydrogen-bonds and π-stacking interactions play a significant role in the biological activity of this series, for example, the hydroxy-oxyindole, phenyl and oxadiazole rings. However, the exact role of each functionality in terms of ligand–target complexes could only be disclosed after the discovery and structural resolution of the molecular target. In addition to the activity prediction, the generated 2D and 4D contribution maps provided information about structural and conformational features that can be used as a valuable tool to guide future efforts in the design of antileishmanial agents.

## Figures and Tables

**Table 1:** The most statistically significant models generated by AutoQSAR using the complete dataset.

Training Set (%)	*R* ^2^	SD	*Q*^2^ (*R*^2^_pred_)	RMSE	N	Fingerprint
70	0.5378	0.2180	0.4937	0.2123	1	Radial
75	0.5997	0.2065	0.5284	0.1994	2	Dendritic
80	0.6304	0.2022	0.6107	0.1817	5	MOLPRINT 2D

*R*^2^: coefficient of determination for the training set; SD: standard deviation; *Q*^2^: predictive correlation coefficient for the test set (*R*^2^_pred_); *RMSE*: root-mean-square error for the test set predictions; N: optimum number of components.

**Table 2:** Statistically significant models generated by AutoQSAR for the G_1_ group.

Training Set (%)	*R* ^2^	SD	*Q*^2^ (*R*^2^_pred_)	RMSE	N	Fingerprint
70	0.8982	0.1178	0.7132	0.1018	2	Radial
75	0.8012	0.1413	0.7022	0.1668	1	Radial
80	0.9069	0.1039	0.8201	0.0945	2	Radial

**Table 3:** Statistically significant models generated by AutoQSAR for the G_2_ group.

Training Set (%)	*R* ^2^	SD	*Q*^2^ (*R*^2^_pred_)	RMSE	N	Fingerprint
70	0.6109	0.205	0.4206	0.1829	2	MOLPRINT 2D
75	0.5693	0.2040	0.5351	0.1041	2	MOLPRINT 2D
80	0.8206	0.1377	0.8001	0.1081	3	Dendritic

**Table 4:** Experimental, predicted, and residual values of pIC_50_ for the G_1_ and G_2_ groups.

		2D-QSAR					4D-QSAR			
		Complete Set Model			Cluster Model		Complete Set Model		Cluster Model	
No.	pIC_50 exp_	pIC_50 pred_	Residue	Group	pIC_50 pred_	Residue	pIC_50 pred_	Residue	pIC_50 pred_	Residue
**1**	5.138	5.184	0.046	G_1_	5.187	0.049	5.066	−0.071	5.21	0.072
**2**	4.913	4.911	−0.002	- ^1^	- ^1^	- ^1^	4.9239	0.011	- ^1^	- ^1^
**3**	5.133	4.962	−0.171	G_1_	5.061	−0.072	5.178	0.045	5.171	0.039
**4**	5.478	5.312	−0.165	G_1_	5.433	−0.044	5.099	−0.379	5.229	−0.249
**5**	4.922	4.955	0.033	G_1_	4.995	0.073	4.848	−0.073	4.893	−0.028
**6**	5.29	5.017	−0.273	G_1_	5.173	−0.116	5.257	−0.033	5.155	−0.135
**7**	5.428	5.231	−0.197	G_1_	5.385	−0.043	5.118	−0.309	5.462	0.035
**8**	4.984	5.103	0.119	G_1_	4.993	0.009	5.194	0.211	5.064	0.081
**9**	5.387	5.47	0.082	G_1_	5.388	−0.001	5.111	−0.276	5.293	−0.094
**10**	4.955	4.904	−0.051	G_1_	5.098	0.143	4.990	0.035	4.882	−0.073
**11**	5.397	5.467	0.07	G_1_	5.554	0.157	5.214	−0.183	5.349	−0.047
**12**	5.188	5.232	0.043	G_1_	5.282	0.093	5.056	−0.132	5.084	−0.104
**13**	4.289	4.848	0.559	G_1_	4.369	0.080	4.747	0.459	4.359	0.071
**14**	5.293	5.158	−0.135	G_1_	5.345	0.052	4.959	−0.334	5.205	−0.088
**15**	5.088	4.848	−0.24	G_1_	4.807	−0.281	5.271	0.184	5.013	−0.075
**16**	5.248	4.848	−0.4	G_1_	5.104	0.144	5.081	−0.167	5.442	0.195
**17**	4.97	5.007	0.037	G_1_	4.97	0.000	5.095	0.125	4.919	−0.051
**18**	4.931	5.141	0.209	G_1_	4.994	0.062	5.196	0.266	5.238	0.308
**19**	5.313	5.333	0.019	G_1_	5.235	−0.079	5.055	−0.258	5.515	0.202
**20**	5.193	5.062	−0.132	G_1_	5.126	−0.067	5.245	0.052	5.168	−0.024
**21**	5.221	5.333	0.112	G_1_	5.245	0.024	5.138	−0.083	5.309	0.089
**22**	5.455	5.543	0.088	G_1_	5.493	0.038	4.993	−0.462	5.306	−0.148
**23**	5.602	5.53	−0.072	G_1_	5.493	−0.109	5.358	−0.244	5.382	−0.219
**24**	5.314	5.185	−0.13	G_1_	5.264	−0.050	4.918	−0.396	5.294	−0.019
**25**	5.137	4.902	−0.235	G_1_	5.182	0.044	5.123	−0.013	5.133	−0.004
**26**	4.658	4.86	0.202	G_1_	4.716	0.058	4.921	0.264	4.924	0.266
**27**	5.545	5.152	−0.393	G_1_	5.47	−0.075	5.565	0.02	5.551	0.007
**28**	4.587	4.882	0.295	G_1_	4.651	0.064	4.468	−0.119	4.583	−0.004
**29**	5.033	4.812	−0.221	G_2_	4.856	−0.177	4.631	−0.402	4.92	−0.113
**30**	5.115	4.982	−0.133	G_2_	5.146	0.031	4.831	−0.284	4.786	−0.329
**31**	5.084	5.094	0.01	G_2_	5.12	0.036	5.135	0.051	5.040	−0.044
**32**	4.592	4.785	0.193	G_2_	4.841	0.249	4.699	0.108	4.695	0.104
**33**	5.081	4.982	−0.099	G_2_	5.112	0.031	5.193	0.113	5.020	−0.06
**34**	4.976	4.785	−0.192	G_2_	4.907	−0.069	5.18	0.204	4.965	−0.011
**35**	5.096	5.074	−0.022	G_2_	5.269	0.173	5.076	−0.02	5.042	−0.053
**36**	4.932	4.883	−0.049	G_2_	4.891	−0.041	5.188	0.257	5.105	0.173
**37**	5.135	4.857	−0.278	G_2_	4.87	−0.265	5.116	−0.019	5.090	−0.045
**38**	4.981	4.992	0.011	G_2_	4.926	−0.055	5.329	0.348	5.133	0.153
**39**	4.598	4.758	0.16	- ^1^	- ^1^	- ^1^	4.5562	−0.042	- ^1^	- ^1^
**40**	4.279	4.373	0.094	G_2_	4.283	0.004	4.802	0.523	4.289	0.011
**41**	4.426	4.35	−0.076	G_2_	4.34	−0.086	4.523	0.097	4.912	0.487
**42**	5.11	5.137	0.027	G_2_	5.163	0.053	4.873	−0.237	5.013	−0.097
**43**	4.755	4.909	0.155	G_2_	4.644	−0.110	5.055	0.3	4.781	0.027
**44**	4.723	4.83	0.107	G_2_	4.595	−0.128	4.831	0.109	5.009	0.287
**45**	4.358	4.736	0.378	G_2_	4.602	0.244	5.010	0.653	4.589	0.232
**46**	4.985	5.014	0.03	G_2_	4.974	−0.011	4.949	−0.035	4.886	−0.099
**47**	4.988	5.373	0.385	G_2_	5.186	0.198	4.937	−0.05	4.944	−0.043
**48**	4.663	4.874	0.212	G_2_	4.868	0.205	4.912	0.25	4.882	0.22
**49**	4.744	4.925	0.181	G_2_	4.728	−0.016	4.826	0.082	4.795	0.051
**50**	4.92	5.028	0.108	G_2_	4.893	−0.027	5.075	0.155	4.838	−0.082
**51**	5.049	5.05	0.001	G_2_	5.092	0.043	5.108	0.059	5.063	0.015
**52**	4.687	4.753	0.067	G_2_	4.828	0.141	4.895	0.209	4.854	0.168
**53**	4.445	4.608	0.164	G_2_	4.456	0.011	4.948	0.504	4.764	0.319
**54**	5.41	4.933	−0.477	G_2_	5.297	−0.113	5.412	0.002	5.518	0.109
**55**	5.068	4.916	−0.152	G_2_	5.07	0.002	4.870	−0.197	5.149	0.082
**56**	4.94	4.925	−0.015	G_2_	4.978	0.038	4.860	−0.079	4.846	−0.093
**57**	5.623	5.444	−0.18	G_2_	5.455	−0.168	5.477	−0.145	5.596	−0.027
**58**	5.008	4.874	−0.134	G_2_	4.908	−0.100	4.992	−0.016	4.96	−0.044
**59**	5.072	5.094	0.022	G_2_	4.871	−0.201	4.875	−0.196	4.856	−0.215
**60**	5.137	5.193	0.055	G_2_	5.219	0.081	4.900	−0.237	4.911	−0.226
**61**	5.291	5.269	−0.022	G_2_	5.299	0.008	5.069	−0.222	4.945	−0.345
**62**	5.07	4.875	−0.196	G_2_	5.115	0.045	5.027	−0.043	4.978	−0.092
**63**	4.747	4.886	0.139	G_2_	4.741	−0.007	4.917	0.17	4.953	0.207
**64**	5.16	5.12	−0.04	G_2_	5.077	−0.083	5.048	−0.112	5.043	−0.116

- ^1^ Structural outlier.

**Table 5:** Statistically significant 4D-QSAR models.

Dataset	*R* ^2^	RMSE	*Q* ^2^_5-fold_	RMSE_cv_	*R* ^2^_pred_
Complete dataset	0.4599	0.2277	0.4137	0.2412	0.4353
G_1_	0.8033	0.1313	0.6600	0.1716	0.6480
G_2_	0.7005	0.1560	0.6095	0.1701	0.6581

**Table 6:** Structures and pIC_50_ values of the dataset compounds used in the QSAR studies.

No.	Structure	pIC_50 exp_	No.	Structure	pIC_50 exp_	No.	Structure	pIC_50 exp_
**1**	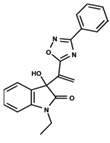	5.138	**2**	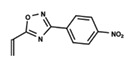	4.913	**3**	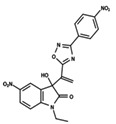	5.133
**4**	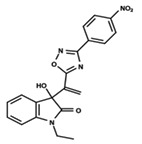	5.478	**5**	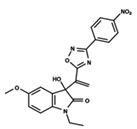	4.922	**6**	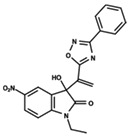	5.29
**7**	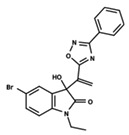	5.428	**8**	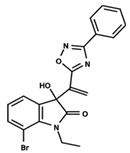	4.984	**9**	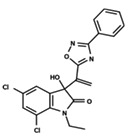	5.387
**10**	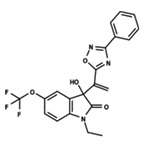	4.955	**11**	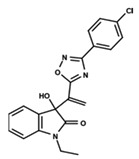	5.397	**12**	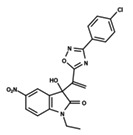	5.188
**13**	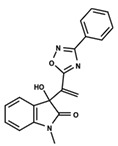	4.289	**14**	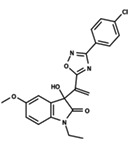	5.293	**15**	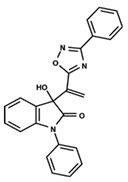	5.088
**16**	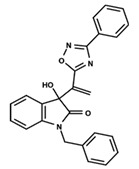	5.248	**17**	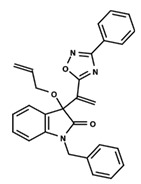	4.97	**18**	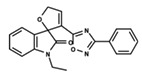	4.931
**19**	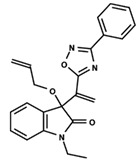	5.313	**20**	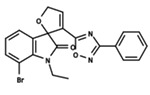	5.193	**21**	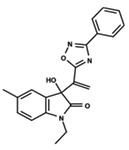	5.221
**22**	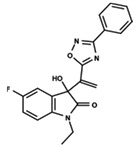	5.455	**23**	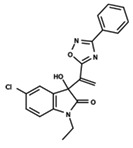	5.602	**24**	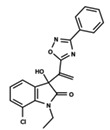	5.314
**25**	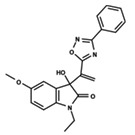	5.137	**26**	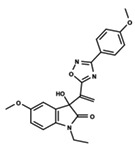	4.658	**27**	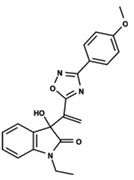	5.545
**28**	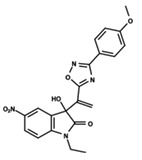	4.587	**29**	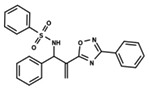	5.033	**30**	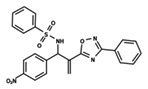	5.115
**31**	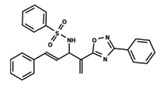	5.084	**32**	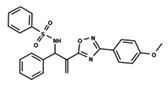	4.592	**33**	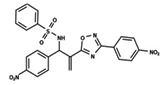	5.081
**34**	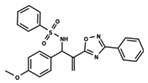	4.976	**35**	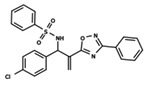	5.096	**36**	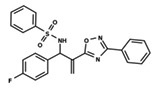	4.932
**37**	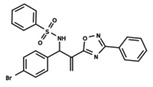	5.135	**38**	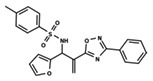	4.981	**39**	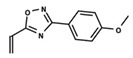	4.598
**40**	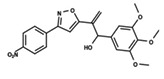	4.279	**41**	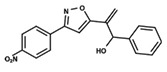	4.426	**42**	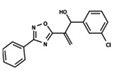	5.11
**43**	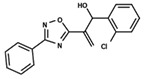	4.755	**44**	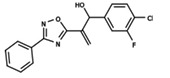	4.723	**45**	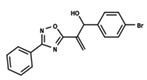	4.358
**46**	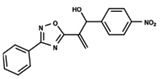	4.985	**47**	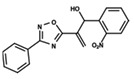	4.988	**48**	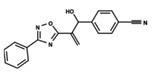	4.663
**49**	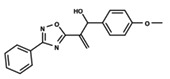	4.744	**50**	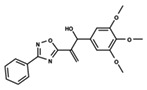	4.92	**51**	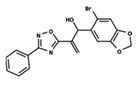	5.049
**52**	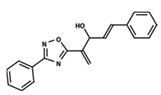	4.687	**53**	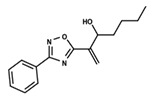	4.445	**54**	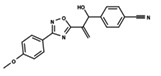	5.41
**55**	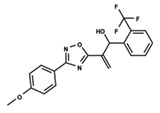	5.068	**56**	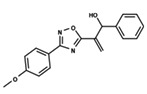	4.94	**57**	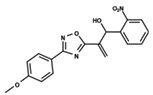	5.623
**58**	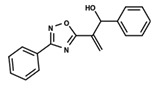	5.008	**59**	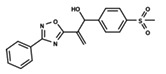	5.072	**60**	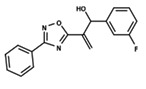	5.137
**61**	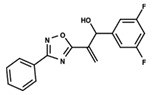	5.291	**62**	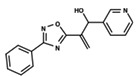	5.07	**63**	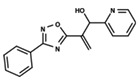	4.747
**64**	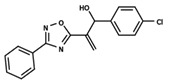	5.16						

## Data Availability

Not applicable.

## Supplementary Materials

The following supporting information can be downloaded at: https://www.mdpi.com/article/10.3390/ijms23168898/s1.
