# Supplementary material for: Hierarchical Clustering and Target-Independent QSAR for Antileishmanial Oxazole and Oxadiazole Derivatives
Source: Int J Mol Sci. 2022 Aug 10;23(16):8898. doi: 10.3390/ijms23168898 (PMC9408707; doi:10.3390/ijms23168898)
Supplement: Supplementary file 1 [file ijms-23-08898-s001.zip › ijms-1806764-Supplementary.pdf]

Supporting Information

Hierarchical Clustering and Target-Independent QSAR for Antileishmanial Oxazole and Oxadiazole Derivatives

Henrique R. Teles [1], Leonardo L. G. Ferreira [1,*], Marilia Valli [1], Fernando Coelho [2] and Adriano D. Andricopulo [1,*]

[1] Laboratory of Medicinal and Computational Chemistry (LQMC), Center for Research and Innovation in Biodiversity and Drug Discovery (CIBFar), Institute of Physics of São Carlos, University of São Paulo (USP), Av. João Dagnone, n° 1100, São Carlos 13563-120, SP, Brazil

[2] Laboratory of Synthesis of Natural Products and Drugs, Institute of Chemistry, University of Campinas, Campinas 13083-970, SP, Brazil

* Correspondence: leonardo@ifsc.usp.br (L.L.G.F.); aandrico@ifsc.usp.br (A.D.A.); Tel.: +55-16-33739844 (A.D.A.)

Results obtained for the QSAR-3D.

For all QSAR-3D calculations, GASTEIGER charges were used. To generate the CoMFA models, the molecules were positioned in a three-dimensional lattice with a spacing of 2Å between the intersections and 4Å of extension in all axes beyond the surface of the molecules. The interaction energy between the atoms of the compounds and the sp3 carbon probe with net charge +1 was estimated with the Tripos force field and the Lennard-Jones and Coulomb potentials. The default value of 30 kcal/mol was used as a cut-off value for both potentials.

Data set	Alignment method	Proportion	R^2	Q^2	SD	Components
All	Rigid-body fit	80x20	0.638	0.335	0.191	3
All	MCS	80x20	0.645	0.246	0.197	3
All	GALAHAD	80x20	0.712	0.118	0.171	4
G_1	Rigid-body fit	80x20	0.749	0.175	0.156	3
G_1	MCS	80x20	0.799	0.293	0.128	3
G_1	GALAHAD	80x20	0.976	0.283	0.058	5
G_2	Rigid-body fit	80x20	0.833	0.038	0.123	3
G_2	MCS	80x20	0.786	0.041	0.150	3
G_2	GALAHAD	80x20	0.903	0.018	0.114	2

MCS: Maximum Common Substructure.
